# High-FOM Temperature Sensing Based on Hg-EIT-Like Liquid Metamaterial Unit

**DOI:** 10.3390/nano12091395

**Published:** 2022-04-19

**Authors:** Jian Li, Yuedan Zhou, Fengwei Peng, Dexu Chen, Chengwei Xian, Pengjun Kuang, Liang Ma, Xueming Wei, Yongjun Huang, Guangjun Wen

**Affiliations:** 1 School of Information and Communication Engineering, Sichuan Provincial Engineering Research Center of Communication Technology for Intelligent IoT, University of Electronic Science and Technology of China, Chengdu 611731, China; lj001@uestc.edu.cn (J.L.); yuedanzhou@163.com (Y.Z.); tonypeng31@163.com (F.P.); chendexu163@163.com (D.C.); xian2002wei@163.com (C.X.); kpj@uestc.edu.cn (P.K.); maliang@uestc.edu.cn (L.M.); keguai2007@163.com (G.W.); 2 Guangxi Key Laboratory of Wireless Wideband Communication and Signal Processing, Guilin University of Electronic Technology, Guilin 541004, China; scuweixue@guet.edu.cn

**Keywords:** liquid metamaterial, temperature sensing, quality factor, figure-of-merit

## Abstract

High-performance temperature sensing is a key technique in modern Internet of Things. However, it is hard to attain a high precision while achieving a compact size for wireless sensing. Recently, metamaterials have been proposed to design a microwave, wireless temperature sensor, but precision is still an unsolved problem. By combining the high-quality factor (Q-factor) feature of a EIT-like metamaterial unit and the large temperature-sensing sensitivity performance of liquid metals, this paper designs and experimentally investigates an Hg-EIT-like metamaterial unit block for high figure-of-merit (FOM) temperature-sensing applications. A measured FOM of about 0.68 is realized, which is larger than most of the reported metamaterial-inspired temperature sensors.

## 1. Introduction

Electromagnetic metamaterials are a kind of artificial sub-wavelength composed of structural materials, with novel electromagnetic properties not found in nature [1,2,3,4,5]. Most of the electromagnetic metamaterials have intrinsic, strong resonance features, with a narrow response frequency band and a high-quality factor (Q-factor) [6,7], making them unsuitable for wide-band electromagnetic wave manipulation applications [8,9,10,11,12]. On the other hand, due to the high electromagnetic resonance strength and high sensitivity to changing background parameters [13,14,15,16], electromagnetic metamaterials with physical, chemical, and biological tunable abilities have been widely used to design various high-performance sensors [17,18,19,20,21,22,23,24,25,26,27,28]. Until now, the reported metamaterial-inspired sensors included physical sensors [19,20,23,25,26,27,28], chemical sensors [21,22], and biological sensors [17,18,24], etc.

For the various metamaterial-inspired sensors reported, sensing sensitivity and precision are two vital parameters for high-performance applications. Unfortunately, most of the reported metamaterial-inspired sensors [17,18,19,20,21,22,23,24], including the reported temperature sensors [25,26,27,28], are based on effective dielectric permittivity, permeability, and/or refractive index changes in the substrates that are used to construct the metamaterials under varying background parameters. To realize more sensitive and higher-precision metamaterial-inspired sensors, researchers have proposed a kind of liquid metamaterial unit [29,30,31,32,33,34] based on fluid metals, such as mercury (Hg) [29,30,35,36], EGaIn, and/or Galinstan [34,37,38]. For the liquid metal-based metamaterial sensors, the electromagnetic response changes are directly from the effective electric length of the metamaterial unit itself, thus allowing for maximum sensitivity to be achieved [35,36]. On the other hand, to obtain a higher precision-sensing performance, the parameter called figure-of-merit (FOM) should be carefully considered. Such FOM is widely defined as

(1) $$\mathrm{FOM}=\frac{\mathrm{Sensitivity}}{3\mathrm{dB}-\mathrm{Bandwidth}}$$

In the above equation, the unit of sensitivity for the temperature sensor discussed in this paper is Hz/°C and the unit of a 3 dB bandwidth is Hz, and so the unit of the defined FOM should be /°C.

From Equation (1), it follows that the designed metamaterial unit should have as narrow a bandwidth as possible, equivalent to a higher Q-factor. In recent years, many kinds of high Q-factor metamaterial units have been reported, including the Fano resonator [39,40], the toroidal resonator [41,42], the anapole resonator [43,44], the FP cavity resonator [45], and the electromagnetically induced transparency (EIT) resonator [46]. Parts of these have been proposed for high-precision sensing applications [17,18,19,35,36]. However, it is very hard to maintain high sensitivity and high precision and, at the same time, have a stable and large, linear sensitivity range for those reported works. In this paper, in contrast to previously reported liquid metal-based metamaterial sensors [35,36] and high Q-factor solid metamaterial sensors [17,18,19], we propose a novel, liquid metal-based EIT-like metamaterial unit block for the high-FOM temperature-sensing applications. The mechanism analysis operating to achieve high sensitivity and high precision, the numerical investigations and optimizations, and the experimental demonstrations used to achieve high-FOM temperature sensing in a large linear range are presented in this paper. Based on a detailed investigation, the proposed liquid metal based EIT-like metamaterial sensor shows better sensing performance, including sensitivity and precision, compared with the previously reported sensors, especially the previously reported temperature sensors, and can be widely used for temperature sensing in the near future.

## 2. Structure Design and Numerical Investigations

### 2.1. Operating Mechanism

**Figure 1:**
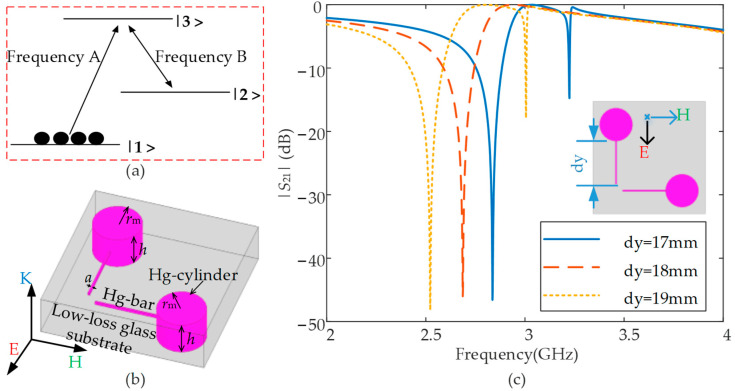
(**a**) Theoretical presentation of the EIT operating mechanism, (**b**) the 3D view of the designed Hg-EIT-like metamaterial unit with parameter definitions, and (**c**) transmission curves of the Hg-EIT-like metamaterial unit under different Hg bar lengths obtained by numerical simulations in the period boundary condition.

According to the theory, electromagnetically induced transparency (EIT) is a kind of quantum interference effect [46]. The basic principle is that when a coherent electromagnetic wave acts on a multi-level atomic system, the atoms will have a strong response to the incident electromagnetic wave with resonance frequency, resulting in the phenomenon of absorption or enhancement. A typical three-level quantum interference system is shown in Figure 1a. If an incident electromagnetic wave can make the atom in the ground state 1> transition to the excited state 3> (the transition frequency is A), the electromagnetic field at this time will be absorbed. At the same time, another incident electromagnetic wave can make an atom with an energy level in the ground state 2> also transition to the excited state 3> (the transition frequency is B), and the ground state 1> and ground state 2> of the atom will be coherent, resulting in the EIT phenomenon.

Based on this theory, in 2018, some researchers designed an EIT-like metamaterial [46], in which the metal wires along the direction of the electric field would be excited under the action of electromagnetic waves to form a bright-state mode, while the parallel bimetallic wire structure along the direction of the magnetic field would have difficulty being excited by an external electric field, thereby forming a dark state mode. The excited, bright-mode metal wire structure radiates electromagnetic waves of a specific frequency outward, thereby exciting the parallel bimetallic wire structure along the direction of the magnetic field. Therefore, both of them are in an excited state, and the radiated electromagnetic waves interfere and cancel each other, reducing the radiation loss and generating a higher Q-factor. Moreover, a novel artificial electromagnetic resonance structure with higher Q value can finally be realized.

### 2.2. Initial Strucure Design and Numerical Anslysis

Based on the low radiation loss working principle of the EIT-like metamaterial, Figure 1b shows the preliminary design of the Hg-based EIT-like metamaterial unit block. Compared with the original structure of a solid state EIT-like metamaterial [44], the newly designed Hg-EIT-like liquid metamaterial unit can achieve low radiation loss as well, while making full use of a large liquid storage structure that can achieve high temperature-sensing sensitivity [33,34], thereby attain a high Q-factor EIT-like resonance mode. In addition, the Hg-EIT-like liquid metamaterial unit can maintain stable temperature-sensing characteristics when it is thermally deformed. The initial simulation results of the electromagnetic wave transmission characteristic curves produced by Ansys HFSS software under different Hg bar lengths are shown in Figure 1c. In such simulations, the periodic boundaries along the *E*- and *H*-directions and Floquet port excitations along the *k*-direction are applied to the free space condition, and the following structural parameters are used: *a* = 0.4 mm, *r*_m_ = 5 mm, and *h* = 6 mm. The dielectric constant and loss tangent of the used glass are *ε*_r_ = 3.7, and tan*δ* = 0.0001, respectively, and the conductivity of Hg is 1.04 × 10^6^ S/m. Moreover, the solving precision tolerance of 0.005 and the interpolating frequency sweep type are defined in the HFSS software to ensure the results are more accurate.

**Figure 2:**
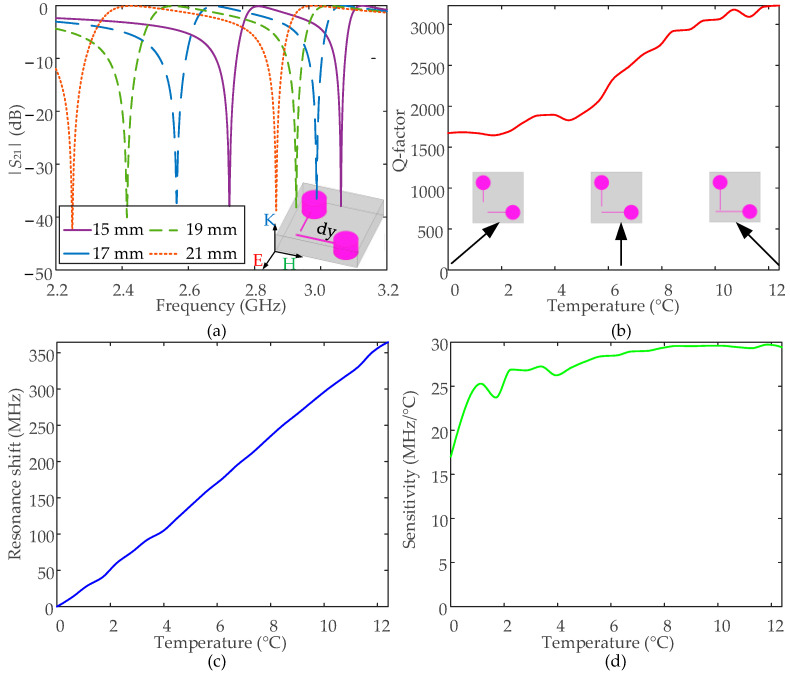
(**a**) The simulated transmission curves of the Hg-EIT-like liquid metamaterial unit under different parameter Hg bar lengths, and (**b**) the calculated Q-factor, (**c**) resonant frequency shift, and (**d**) temperature sensing sensitivity as a function of changes in temperature.

Specifically, the more precise simulation results are shown in Figure 2, including the transmission curves under wider Hg bar changing range, the calculated Q-factors, the resonance frequency shit, and the sensitivity as a function of temperature. Figure 2b shows that the Hg-EIT-like liquid metamaterial unit has a high resonance Q-factor, but the Q-factor fluctuates to a certain extent with changes in temperature.

In Figure 2, the temperature is theoretically calculated based on the thermal expansion rate of the liquid metal Hg, *γ* = (1/*V*_0_)·(∆*V*/∆*T*). Here, *V*_0_ is the initial total volume of the Hg-EIT-like resonator shown in Figure 1b, ∆*V* is the volume-change amount at temperature change ∆*T*. Specifically, based on the structure shown in Figure 1b, the total Hg volume is

(2) $$V_{0}=2\pi r_{m}{}^{2}h+a^{2}(d_{y}+d_{x})$$

where *r_m_* and *h* are the radius and height of the Hg cylinder, *a* is the side length of the cross section of the square Hg bar, and *d_y_* and *d_x_* are the Hg bar lengths along the *y*-axis and the *x*-axis. Therefore, the relationship between the Hg bar length changes and the temperature changes calculated from the thermal expansion coefficient of Hg is

(3) $$\Delta l=\frac{\Delta V}{2a^{2}}=\frac{V_{0}\Delta T\gamma }{2a^{2}}$$

The Hg bar length at the initial temperature of the Hg-EIT-like liquid metamaterial unit can be set as =15 mm, and therefore, the initial Hg volume is around 788 mm^3^. Figure 2b–d shows that, when the temperature change is 12.4 °C, the resonant frequency offset of the Hg-EIT-like liquid metamaterial unit reaches 370 MHz, and the temperature-tuning sensitivity is about 17–30 MHz/°C in the temperature-tuning range. However, as mentioned before, the temperature-sensing sensitivity is not stable enough, and the relative change reaches 88%.

### 2.3. Structure Optimizations

**Figure 3:**
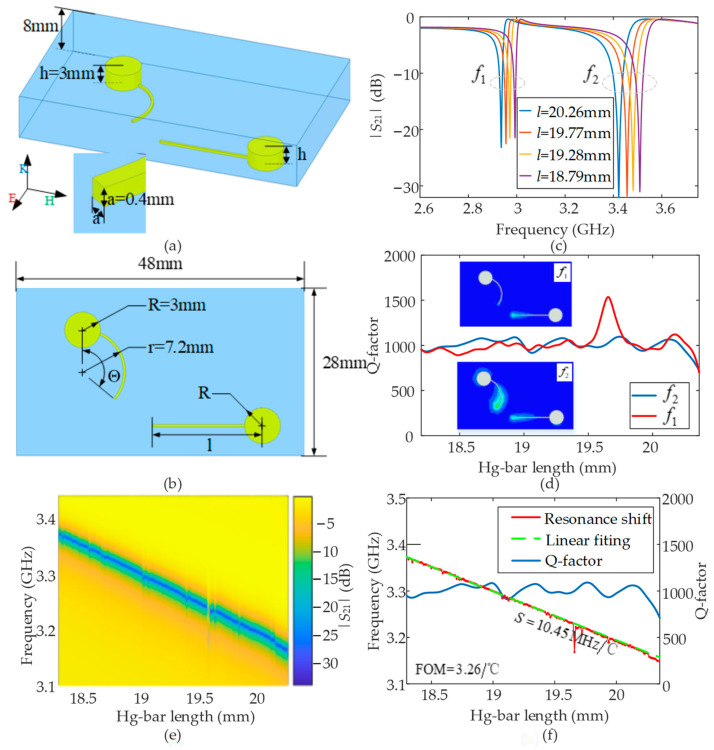
(**a**,**b**) The 3D and 2D views of the modified Hg-EIT-like metamaterial unit with parameter definitions; (**c**) the transmission curves under different Hg bar lengths obtained by numerical simulations in the period boundary condition; and (**d**–**f**) the simulated and calculated transmission 2D spectrum, the Q-factor, and the sensitivity results. The insets of panel (**d**) are the electric field distributions at the two resonance frequencies.

As mentioned in the previous subsection, the initially obtained temperature-sensing performance is not stable in terms of the Q-factor and the sensitivity under different temperature condition, as shown in Figure 2. Based on the bright-mode and dark-mode coupling mechanism for the EIT-like metamaterial resonator, the coupling strength should be kept near-constant at all structural sizes, especially the Hg bar lengths for the Hg-EIT-like liquid metamaterial unit designed in this paper. However, the original configuration shown in Figure 1 cannot satisfy such a requirement. In order to strengthen the performance stabilities of both the electromagnetic resonance characteristics and the temperature-tuning ability for the Hg-EIT-like liquid metamaterial unit, the original structure shown in Figure 1b continues to be improved and optimized to meet the practical application requirements of high-performance temperature sensing, and the new structure is shown in Figure 3a,b. For this new configuration, when the Hg bar length is increased under temperature changes, the coupling strength between those two bars can be maintained properly so that the Q-factor should be more stable. Specifically, in order to ensure sufficient coupling of the two resonant arms under different lengths, one resonator arm is designed as a circular arc and the other resonator arm is designed as a straight line. The Hg injection channel communicates with the outside. When the temperature of the structure changes, both the arc-shaped vibrating arm and the straight resonating arm are correspondingly elongated or shortened, so that the structure has good temperature-control reconfigurability and temperature-control sensitivity. After simulation optimization, the size parameters of the structure are determined. As shown in Figure 3, the basic low-loss glass structure is 48 mm long and 28 mm wide; the Hg storage cylinder radius is 8 mm high, and the thickness is 8 mm; the arc radius of the arc resonance arm is 3 mm; height is 3 mm; the thickness of the glass is 8 mm; the corresponding arc angle is *θ*; the length of the straight resonant arm is *l*; and the length of the square side of the cross section of the resonant arm is 0.4 mm.

Based on the above structural parameter definitions, the corresponding relationship between the total volume, the Hg bar length, and the temperature of the improved new Hg-EIT-like liquid metamaterial unit is represented as follows:

(4) $$V_{1}=\pi R^{2}h+2\pi Ra^{2}\left(\frac{\theta }{360}-\frac{\arcsin(R/2r)}{180}\right)$$

(5) $$V_{2}=\pi R^{2}h+a^{2}\left(l-2\sqrt{R^{2}-{\left(\frac{a}{2}\right)}^{2}}\right)-a\left(\frac{\arcsin\left(a/2R)\right)}{180{}^{\circ}}\pi R^{2}-\frac{a}{2}\sqrt{R^{2}-{\left(\frac{a}{2}\right)}^{2}}\right)$$

(6) $$\Delta \theta =\frac{\Delta V_{1}\cdot 180}{a\pi \left(r^{2}-{\left(r-a\right)}^{2}\right)}=\frac{V_{1}\cdot \Delta T\cdot \gamma \cdot 180}{a\pi \left(r^{2}-{\left(r-a\right)}^{2}\right)}$$

(7) $$\Delta l=\frac{\Delta V_{2}}{a^{2}}=\frac{V_{2}\cdot \Delta T\cdot \gamma }{a^{2}}$$

In the above equations, *V*_1_ is the volume of the arc-shaped Hg column structure in the figure, and *V*_2_ is the volume of the straight Hg column structure. The arc-shaped part and the straight Hg bar can both produce elongation and shortening effects with the change in temperature. The arc-shaped structure corresponds to the circumference angle *θ*, and the straight Hg bar length is represented by *l*. Δ*θ* is the variation in the circumferential angle of the arc part, Δ*V*_1_ is the volume change in the length of the straight Hg bar, Δ*V*_2_ is the change in the arc part, and Δ*T* is the amount of change in the temperature of the entire liquid metal Hg. According to the improved Hg-EIT-like liquid metamaterial unit and the corresponding relationship between the structure and temperature shown in Equations (4)–(7), the initial circumferential angle of the arc-shaped mercury column structure is set as 50 deg; the initial length of the straight Hg bar structure is 18.3 mm; and thus, the calculated Δ*θ* is about 0.4 deg/°C and Δ*l* is about 0.1 mm/°C.

Here, we further use HFSS software to simulate the transmission coefficients of the structure under four resonant arm length sizes, as shown in Figure 3c. Here, the period boundary condition and Floquet port excitation are also applied to the unit cell. We see that, under the four size conditions, the structure produces two resonance peaks. Taking the size of the straight resonant arm 18.79 mm as an example, the electric field distributions of the simulated structure at the frequencies of the two resonance peaks are shown in the inset of Figure 3d. As can be seen, the arc-shaped resonator arm is excited by the electric field to generate a bright mode, and the straight resonator arm perpendicular to the direction of the electric field cannot be directly excited by the electric field into a dark mode. Consequently, the electric field is coupled back and forth between the dark mode and the bright mode. At this time, the electric field energy is mainly concentrated around the direct resonance, resulting in an EIT-like resonance mode. While the structure is at the second resonance peak frequency, the direct resonance arm is still excited and a strong electric field is generated around the resonant arm and the arc part. At the same time, the electromagnetic wave energy is bound around the straight resonator arm and the arc resonator arm, resulting in a greater resonance intensity.

Through the numerical simulation, the resonance Q-factor of the structure under the different resonant arm lengths is obtained, as shown in Figure 3d. The resonance Q-factor of the first resonance peak can be seen to range from 893 to 1500 and the Q-factor of the second resonance peak ranges from 924 to 1090. The resonance strength of the second resonance peak is thus better than that of the first resonance peak. Therefore, the second resonance peak was selected for the follow-up study. Specifically, we took the straight resonant arm to change ~9.8 μm, and the arc resonator arm to change 0.04 deg. This setting is analogous to the resonant characteristic changes of the structural unit with the temperature changing step of 0.1 °C and the variation range of 20 °C. The transmission coefficient curve spectrum of the improved Hg-EIT-like liquid metamaterial unit with different lengths is obtained, as shown in Figure 3e. At the same time, according to the simulation results, the theoretical linear function of the resonant frequency of the structural unit and the length of the resonator arm are fitted to the simulation data, as shown in Figure 3f. The figure shows that the temperature-sensing sensitivity of the improved Hg-EIT-like liquid metamaterial unit obtained by the simulation has an average Q-factor of about 1000 and a FOM value of 3.26/°C.

## 3. Experimental Demonstrations

**Figure 4:**
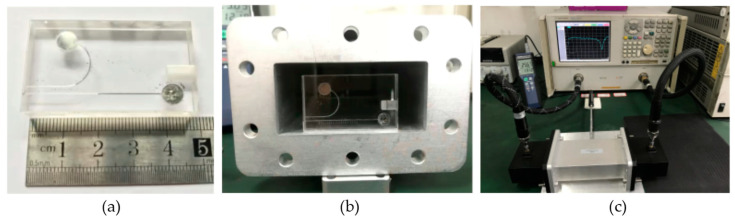
(**a**) Fabricated and assembled Hg-EIT-like metamaterial unit prototype, (**b**) the simple under test placed in the rectangular waveguide, and (**c**) the whole measurement setup.

Based on the simulation optimization results and the parameters obtained above, in this section, we use the silica glass as the low-loss transparency substrate to fabricate the Hg-EIT-like liquid metamaterial unit. The steps of processing, bonding, and packaging are shown as follows: (1) using a laser-etching technology to etch the EIT-like structure channel shown in Figure 3a on the silica glass base cube; (2) using a UV curing glue to paste another thin glass sheet onto the glass substrate structure engraved with the EIT-like structure channel; (3) using a UV lamp to curve and encapsulate the bonded two-layer glass structure; and (4) injecting the liquid metal Hg into the cylindrical liquid storage tank and the Hg bar channel along the injection flow channel using tools such as thin syringes and needle tubes. Finally, the processed Hg-EIT-like liquid metamaterial unit is shown in Figure 4a.

**Figure 5:**
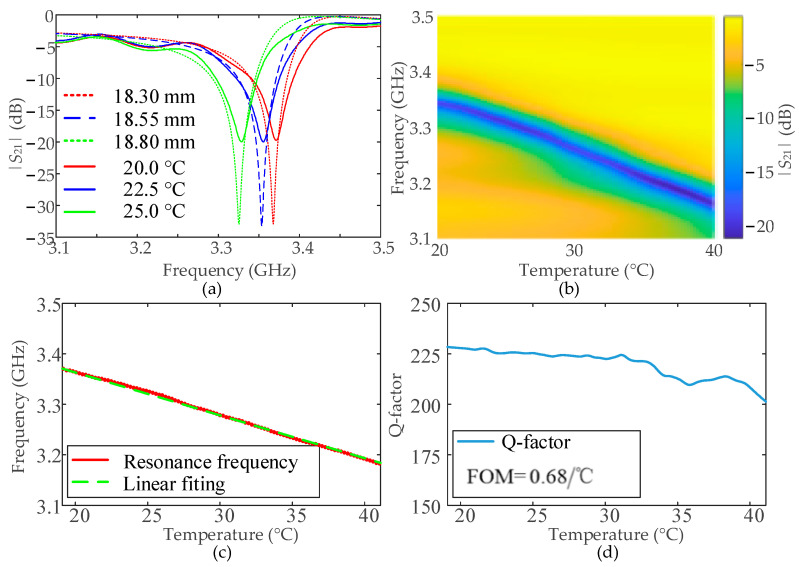
(**a**) Simulated and measured |*S*_21_| curves at different Hg bar lengths and temperature values, respectively, and (**b**) measured transmission 2D spectrum for the fabricated Hg-EIT-like metamaterial unit; (**c**,**d**) the collected resonance frequency shift and calculated Q-factor, and FOM.

Next, the prepared Hg-EIT-like liquid metamaterial unit is placed in the center of the standard-size rectangular waveguide BJ32 (as shown in Figure 4b, the waveguide operating frequency range: 2.60–3.95 GHz), and the waveguide is connected to the vector network analyzer (Agilent 5230A). The closed rectangular waveguide used in this paper can help to concentrate the incident electromagnetic wave [47,48], resulting in smaller radiation loss and so a higher Q-factor. This will contribute to a stable and better FOM (Equation (1)) by controlling the ambient temperature to slowly increase from 20 °C to 40 °C (as shown in Figure 4c) with a temperature-changing step of 0.1 °C. The electromagnetic structure transmission coefficient curves obtained by the vector network analysis are summarized in Figure 5a,b. The extracted electromagnetic resonance frequency and the resonance Q-factor changes with increases in temperature are shown in Figure 5c,d. At the same, the corresponding numerical simulations of the same conditions are shown in Figure 5a for comparison. The figure shows that the measured |*S*_21_| curve at each temperature well matches the simulated result at the corresponding Hg bar length, especially the resonance frequency. The measured resonance strength is smaller than the simulated one, which is due to the unperfect metamaterial unit fabrication and the Hg-injection process, resulting in additional loss. Moreover, the actual glass container and rectangular waveguide used will contribute more electromagnetic loss.

As shown in Figure 5b, when the temperature increases from 20 °C to 40 °C, the measured resonant frequency of the Hg-EIT-like liquid metamaterial unit decreases from 3.37 GHz to 3.18 GHz, and its average Q-factor is about 210. The electromagnetic resonance frequency of the improved Hg-EIT-like liquid metamaterial unit has an almost linear relationship with the rise in temperature. By comparing the simulation results of the unit structure in Figure 3 to the test results in Figure 5, the Q-factor obtained by the test can to seen to be lower than the simulation result. This is because the unit structure placed in the test process does not fully fill the entire rectangular waveguide, and the electromagnetic leakage causes its Q-factor to drop.

## 4. Discussion

Table 1 summarizes the simulated and measured performance parameters of the previously reported liquid metal-based temperature-sensitivity metamaterial unit [35,36] and some other temperature-sensing techniques [49,50,51,52] with the proposed structure in this paper, including the temperature-sensing sensitivity, the resonant Q-factor, and the FOM. First, it can be seen that the average Q-factors obtained by the actual tests of the three Hg structures are all smaller than the corresponding simulation results. After the analysis, as mentioned previously, we found two main reasons. One is the difference between the rectangular waveguide in the simulation environment and the actual test environment. Second, there is a difference between the electromagnetic parameters of the structural material and the prototype assembling actually processed in measurements, and the electromagnetic parameters of the structure used in the simulations. The temperature-sensing sensitivity obtained by the test is close to the simulations for all three designs. However, among them, the EIT-like structure has the highest measured Q-factor, even though the temperature-sensing sensitivity is the lowest one. As analyzed in the previous section, the sensitivity can be enhanced by simply enlarging the Hg cylinder tank based on the theory of the thermal expansion rate of the liquid metal Hg. As shown in Table 1, because the EIT-like metamaterial unit can achieve the maximum measured Q-factor, the calculated FOM is thus the largest one compared with other two designs. Therefore, the best temperature-sensing precision is achieved, and it can be further enhanced by increasing the Hg cylinder tank volume and by reducing the Hg bar cross-section. However, as shown in the Hg-EIT-like metamaterial unit configuration, the Hg-bar length enhanced range is limited due to the limited space in the glass tube. Additionally, if the Hg bar is increased too much, the EIT-like resonance with high Q-factor performance cannot be kept well. Therefore, the good sensing sensitivity and precision performances for the proposed Hg-EIT-like metamaterial unit can be only worked at a limited sensing range. For that reason, it is preferable to use this discussed temperature sensor for some of the high-precision temperature-sensing areas with limited temperature varying range.

Moreover, most of the other reported sensors [49,50,51,52] are achieved by injecting the temperature-sensitive liquid dielectric materials, including Benzene, specific liquid material (LM), dimethyl sulfoxide (DS), and liquid crystal (LC), into the single/dual cores of the photonic crystal fibers. In this way, the sensitivity and FOM can be obtained by detecting the changes in resonance wavelength induced by the the variations in temperature. As can be seen, even though those sensors show higher sensitivity at the optical wavelength, the achieved FOM is much smaller than the Hg-inspired metamaterial sensor configuration, due to the poor optical resonance Q-factors.

The near-linear temperature-sensing results obtained in the foregoing discussions are limited to the low-power incident electromagnetic wave condition. However, if the incident wave power is increased, some new phenomena could arise, which may affect the sensing performance. First, in contrast to the conventional metamaterial unit, which is constructed with solid-state metals such as copper, the liquid metamaterial unit discussed in this paper composed of Hg has a larger ohm loss because of the conductivity of Hg being 1.04 × 10^6^ S/m, which is one order magnitude smaller than copper. Consequently, if the high power, incident electromagnetic waves acted on the Hg-EIT-like structure, parts of the energy will be transferred into the structure as ohm heating and, as a result, the Hg bar length will be increased even though there are no background environmental changes in temperature. This is a complex, nonlinear dynamic process within the metamaterial unit [53,54,55]. In that case, the temperature-sensing sensitivity will be affected slightly and the real background temperature cannot be measured correctly. Therefore, to maintain the near-linear sensing performance, low-power incident electromagnetic wave should be used in this temperature sensor based on the Hg-EIT-like metamaterial unit.

On another hand, due to the interaction between the incident electromagnetic wave energy and the thermal energy within the Hg metamaterial unit mentioned above, this designed high Q-factor Hg-EIT-like structure can also be used as an electromagnetic field strength sensor [56,57].

## 5. Conclusions

This paper proposes for high-FOM temperature-sensing applications a novel EIT-like metamaterial unit based on the liquid metal Hg with large temperature-sensing sensitivity and precision. It discusses the detailed design for a high-performance Hg-EIT-like metamaterial unit and provides the corresponding numerical simulation and optimizations, as well as the experimental demonstrations to verify the proposed design. A measured FOM of about 0.68 is realized, which is larger than most of the reported metamaterial inspired temperature sensors. The proposed Hg-EIT-like metamaterial unit can be widely used for high-performance temperature sensing in the future.

## Figures and Tables

**Table 1:** Performance comparisons for the proposed temperature sensor structures.

Resonant Structure	Sensitivity		Q-Factor		FOM (/°C)	
	Sim.	Mea.	Sim.	Mea.	Sim.	Mea.
Fano [35]	15.7 MHz/°C	16.4 MHz/°C	~800	~80	~6.3	~0.59
Anapole [36]	16 MHz/°C	17.14 MHz/°C	~230	~65	~1.53	~0.3
Benzene-PCF [49]	12 nm/°C	–	~90	–	~0.4	–
LM-PCF [50]	2.15 nm/°C	–	~50	–	~0.061	–
DS-PCF [51]	–	5.55 nm/°C	–	~70	–	~0.076
LC-PCF [52]	2.82 nm/°C	–	~20	–	~0.048	–
EIT-like	10.45 MHz/°C	9.5 MHz/°C	~1000	~210	~3.26	~0.68

## Data Availability

Data can be available from the authors upon request.
